# Lidocaine alleviates morphine tolerance via AMPK-SOCS3-dependent neuroinflammation suppression in the spinal cord

**DOI:** 10.1186/s12974-017-0983-6

**Published:** 2017-11-02

**Authors:** Yan Zhang, Gao-Jian Tao, Liang Hu, Jie Qu, Yuan Han, Guangqin Zhang, Yanning Qian, Chun-Yi Jiang, Wen-Tao Liu

**Affiliations:** 1 Neuroprotective Drug Discovery Key Laboratory of Nanjing Medical University, Department of Pharmacology, Nanjing Medical University, Nanjing, Jiangsu 211166 China; 2 Research Division of Pharmacology, China Pharmaceutical University, Nanjing, Jiangsu 211100 China; 3 Department of Pain, Affiliated Drum Tower Hospital, Medical School of Nanjing University, Nanjing, Jiangsu 210008 China; 4 Jiangsu Province Key Laboratory of Anesthesiology, Xuzhou Medical University, Xuzhou, Jiangsu 221004 China; 5 Department of Anesthesiology, The First Affiliated Hospital of Nanjing Medical University, Nanjing, Jiangsu 210029 China; 6 Department of Pharmacy, Sir Run Run Shaw Hospital Affiliated to Nanjing Medical University, Nanjing, Jiangsu 210008 China

**Keywords:** Morphine tolerance, SOCS3, AMPK, Neuroinflammation, Microglia

## Abstract

**Background:**

Morphine tolerance is a clinical challenge, and its pathogenesis is closely related to the neuroinflammation mediated by Toll-like receptor 4 (TLR4). In Chinese pain clinic, lidocaine is combined with morphine to treat chronic pain. We found that lidocaine sufficiently inhibited neuroinflammation induced by morphine and improved analgesic tolerance on the basis of non-affecting pain threshold.

**Methods:**

CD-1 mice were utilized for tail-flick test to evaluate morphine tolerance. The microglial cell line BV-2 was utilized to investigate the mechanism of lidocaine. Neuroinflammation-related cytokines were measured by western blotting and real-time PCR. The level of suppressor of cytokine signaling 3 (SOCS3) and adenosine 5′-monophosphate (AMP)-activated protein kinase (AMPK)-related signaling pathway was evaluated by western blotting, real-time PCR, enzyme-linked immunosorbent assay (ELISA), and immunofluorescence staining.

**Results:**

Lidocaine potentiated an anti-nociceptive effect of morphine and attenuated the chronic analgesic tolerance. Lidocaine suppressed morphine-induced activation of microglia and downregulated inflammatory cytokines, interleukin-1β (IL-1β), and tumor necrosis factor-alpha (TNF-α) via upregulating SOCS3 by activating AMPK. Lidocaine enhanced AMPK phosphorylation in a calcium-dependent protein kinase kinase β (CaMKKβ)-dependent manner. Furthermore, lidocaine decreased the phosphorylation of p38 mitogen-activated protein kinase (MAPK) and inhibited the nuclear factor-κB (NF-κB) in accordance with the inhibitory effects to TLR4.

**Conclusions:**

Lidocaine as a prevalent local anesthetic suppresses morphine tolerance efficiently. AMPK-dependent upregulation of SOCS3 by lidocaine plays a crucial role in the improvement of analgesic tolerance.

**Electronic supplementary material:**

The online version of this article (10.1186/s12974-017-0983-6) contains supplementary material, which is available to authorized users.

## Background

Morphine is prevalent in treating acute and chronic pain today. However, marked analgesic tolerance along with other adverse effects such as addiction and dependence [1] significantly hinders its application [2], which represents a great challenge due to its unclear mechanism.

Despite intensive research into the neurobiological mechanisms of morphine tolerance, including endocytosis of micro-opioid receptor (MOR), upregulation of *N*-methyl-d-aspartic acid (NMDA) receptor, downregulation of γ-aminobutyric acid (GABA) receptor [3], and activation of protein kinase C (PKC) [4, 5], morphine tolerance is still difficult to manage by current drugs. However, many studies have shown that neuroinflammation especially mediated by Toll-like receptor 4 (TLR4) is a very important reason for the development of tolerance [6–8].

TLR4 recognizes various pathogenic ligands, and its activation upregulates inflammatory cytokines in immune cells [9, 10]. In the central nervous system (CNS), microglia are important TLR4-expressing cells that mediate neuroinflammation. Chronic administration of morphine activates microglia and increases proinflammatory cytokines via TLR4/p38/NF-κB pathway [11, 12]. The proinflammatory cytokines, especially interleukin-1β (IL-1β) and tumor necrosis factor-α (TNF-α), further induce the activation of PKC in neurons, leading to central sensitization and weakened analgesic effect [13]. Previous studies demonstrated that systemic antagonism of TLR4 inhibited the activation of microglia and improved morphine tolerance [14]. Therefore, inhibiting TLR4-mediated neuroinflammation would be an effective strategy for improving morphine tolerance [15]. However, up till now, there is no available safe and efficient drug for suppressing TLR4, IL-1β, and TNF-α in clinical to alleviate tolerance.

Suppressor of cytokine signaling (SOCS) is a family of intracellular proteins with eight family members that negatively regulate the inflammation [16]. Recently, increasing evidences detailing suppressor of cytokine signaling 3 (SOCS3)’s broad-acting regulation in many biological processes implicated its role in immune disorders; thus, SOCS3 was identified as a key protein at the cross roads of numerous intracellular and pathological events [17]. Studies showed the effect of SOCS3 on TLR4 signal pathway based on its ability of inhibiting the activation of TNF receptor-associated factor 6 (TRAF6) and TGF-β-activated kinase 1 (TAK1), both of which are crucial for TLR4 inflammatory responses. Based on these studies, the agents increasing the level of SOCS3 could potentially inhibit TLR4-mediated neuroinflammation.

Lidocaine is a widely used short-acting local anesthetic and anti-arrhythmic agent [18]. Previous studies have demonstrated that low-dose intravenous administration of lidocaine had anti-hyperalgesia and anti-inflammatory effects [19, 20]. Furthermore, lidocaine was also utilized as an immunomodulatory drug in the treatment against allergic diseases [21]. It was also reported that lidocaine could reduce opioid consumption and improve postoperative quality of recovery after ambulatory laparoscopic surgery [22]. Mounting evidences indicated that lidocaine may suppress the activation of microglia and inhibit the subsequent inflammation. Based on these properties, lidocaine is clinically utilized to combine with morphine in the treatment of chronic severe pain in China.

In this study, we found that lidocaine upregulated the level of SOCS3 and phosphorylation of adenosine monophosphate-activated protein kinase (AMPK). Lu et al. reported that bupivacaine, a local anesthetic similar with lidocaine, was able to activate AMPK [23]. Collectively, we considered whether the coordination between SOCS3 and AMPK implicated the molecular mechanism of lidocaine in its anti-inflammatory effects during the treatment of morphine tolerance. Therefore, we hypothesized that the therapeutic effect of lidocaine on the morphine tolerance was dependent on AMPK-induced SOCS3 and we further investigated the mechanism of lidocaine on the regulation of SOCS3.

## Methods

### Animals

Adult male CD-1 mice (18–22 g) were provided by the Experimental Animal Center at Nanjing Medical University, Nanjing, China. Animals were housed five to six per cage under pathogen-free conditions with soft bedding under controlled temperature (22 ± 2 °C) and a 12-h light/dark cycle (lights on at 8:00 a.m.). Behavioral testing was performed during the light cycle (between 9:00 a.m. and 5:00 p.m.). The animals were allowed to acclimate to these conditions for at least 2 days before starting experiments. For each group of experiments, the animals were matched by age and body weight.

### Chemicals and reagents

Lidocaine was purchased from MedChem Express (MCE), USA. Morphine hydrochloride was purchased from Shenyang First Pharmaceutical Factory, Northeast Pharmaceutical Group Company (Shenyang, China). SOCS3 small interfering RNA (siRNA, m) and control siRNA were purchased from Santa Cruz Biotechnology (Santa Cruz, CA, USA). STO-609 was purchased from Tocris Bioscience (Bristol, UK). Compound C, BAPTA-AM, and ethylene glycol tetraacetic acid (EGTA) were from Sigma-Aldrich (St. Louis, MO, USA). Ca^2+^ fluorescence dye Fluo-4 was purchased from Life Technologies (Grand Island, NY, USA). Ca^2+^ fluorescence dye Fluo-2 was purchased from KenGEN (KenGEN BioTECH, China). Antibody for β-actin was from Sigma-Aldrich (St. Louis, MO, USA). Antibody for SOCS3 was from Abcam (Cambridge, MA, USA). Antibody for suppressor of cytokine signaling 1 (SOCS1) was from Santa Cruz Biotechnology (Santa Cruz, CA, USA). Antibodies for p38, phosphorylated AMPK (Thr172), phosphorylated p38 (Tyr182), phospho-NF-κB p65 (Ser536), NF-κB p65/RelA, TNF-α, and p-protein kinase A (PKA, Thr197) were from Cell Signaling Technology (Beverly, MA, USA). Antibody for IL-1β was purchased from R&D Systems (Minneapolis, MN, USA). Secondary antibodies were from Sigma-Aldrich (St. Louis, MO, USA). Immunofluorescent antibody for glial fibrillary acidic protein (GFAP) and neuronal nuclear protein (NeuN) were from Millipore (Billerica, MA, USA). Immunofluorescent antibody for ionized calcium-binding adapter molecule 1 (Iba1) was from Abcam (Cambridge, MA, USA). Immunofluorescent antibodies for c-Fos and calcitonin gene-related peptide (CGRP) were from Cell Signaling Technology (Beverly, MA, USA). Secondary antibodies were from Jackson ImmunoResearch Laboratories (West Grove, PA, USA). Fetal bovine serum (FBS) was purchased from Gibco, and other cell culture media and supplements were purchased from KenGEN (KenGEN BioTECH, China). All other reagents were from Sigma-Aldrich (St. Louis, MO, USA).

### Tolerance models and behavioral analysis

Animals were habituated in the testing environments for 2 days, and behavioral testing was carried out in a blinded manner. Lidocaine was suspended with 1 μg/μL morphine. For the test of chronic tolerance, mice were intrathecally injected with saline or morphine (10 μg/10 μL) once daily for seven consecutive days with or without lidocaine (100, 200, and 400 μg/10 μL). Behavioral testing was performed 1 h after morphine administration by tail-flick assay every morning. Briefly, mice’s tails were placed in 52 °C water, and the latency of tail withdrawal was measured. A cutoff time of 10 s was set to avoid tissue damage. Data were calculated as a percentage of maximal possible effect (%MPE), which was calculated by the following formula: 100% × [(Drug response time − Basal response time) / (10 s − Basal response time)] = %MPE.

### Intrathecal injection procedure

To perform intrathecal (i.t.) injections, the mice were placed in a prone position and the midpoint between the tips of the iliac crest was located. A Hamilton syringe with a 30-gauge needle was inserted into the subarachnoid space of the spinal cord between the L5 and L6 spinous processes. Proper intrathecal injection was systemically confirmed by observation of a tail flick. Intrathecal injection did not affect baseline responses, compared with latencies recorded before injection.

### Cell cultures

BV-2 cells were maintained in humidified 5% CO_2_ at 37 °C in Dulbecco’s modified Eagle’s medium (DMEM; KenGEN BioTECH, China) supplemented with 10% (*v*/*v*) FBS (Gibco), 80 U/mL penicillin, and 0.08 mg/mL streptomycin. For further experiments, 10^5^ cells were plated in a 6-well plate or 12-well plate overnight and then treated with morphine (200 μM) in the following morning with or without lidocaine for 12 h. Cell extracts and precipitated supernatants were analyzed by immunoblot assay or real-time PCR.

### NF-κB activation assay

BV-2 cells were plated in glass bottom cell culture dishes and treated with morphine (200 μM) for 1 h with or without lidocaine (10 μM). Then, BV-2 cells were fixed with 4% paraformaldehyde and were permeated with 0.3% Triton X-100. After blocking with 10% donkey serum in phosphate-buffered saline (PBS) for 2 h, the coverslips with BV-2 cells were incubated at 4 °C with the p65/RelA antibody diluted in PBS (1:200) overnight. Then, the coverslips were exposed to the fluorescein isothiocyanate (FITC)-conjugated anti-rabbit IgG (1:300, at room temperature for 1 h) and then were rinsed three times with PBS. Finally, the coverslips were stained with 1 μg/mL 4′,6-diamidino-2-phenylindole (DAPI, a fluorescence DNA dye to mark the nucleus) for 1 min. Confocal microscopy analysis was carried out using a Carl Zeiss LSM710 confocal system.

### Western blot

Samples (cells or spinal cord tissue segments at L1–L6) were collected and washed with ice-cold PBS before being lysed in radio immunoprecipitation assay (RIPA) lysis buffer, and then sample lysates were separated by SDS-PAGE and electrophoretically transferred onto polyvinylidene fluoride membranes (Millipore). The membranes were blocked with 10% milk in TBST (Tris–HCl, NaCl, Tween 20) for 2 h at room temperature and then probed with primary antibodies at 4 °C for overnight. Finally, the horseradish peroxidase (HRP)-coupled secondary antibodies were utilized for detecting corresponding primary antibody. The primary antibodies utilized included β-actin (1:5000), SOCS1 (1:100), SOCS3 (1:1000), p-AMPK (Thr172) (1:1000), p-p38 (Tyr182) (1:1000), p38 (1:1000), p-p65 (Ser536) (1:1000), p65 (1:1000), IL-1β (1:300), TNF-α (1:300), and p-PKA (Thr197) (1:1000). The bands were then developed by enhanced chemiluminescence reagents (PerkinElmer, Waltham, MA, USA). Data were analyzed with the Molecular Imager and the associated software Quantity One-4.6.5 (Bio-Rad Laboratories, USA).

### Immunohistochemistry

Under deep anesthesia by intraperitoneal injection of chloral hydrate (400 mg/kg), the animal was perfused with normal saline followed by 4% paraformaldehyde in 0.1 M PBS, pH 7.2–7.4, for 20 min. Then, L4 and/or L5 lumbar segments were dissected out and post-fixed in the same fixative. The embedded blocks were sectioned as 25 μm in thickness and processed for immunofluorescence assay. Sections from each group (four mice in each group) were incubated with primary antibody: SOCS3 (1:200), Iba1 (1:200), NeuN (1:200), GFAP (1:200), c-Fos (1:200), and CGRP (1:200). Then, the free-floating sections were washed with PBS and incubated with the secondary antibody (1:300) for 2 h at room temperature. After being washed three times with PBS, the samples were investigated with an immunofluorescence microscope (Zeiss AX10, Germany). Images were randomly coded, and the fluorescence intensities of Iba1-, CGRP-, and c-Fos-positive dots were analyzed by Image-Pro Plus 6.0 software (Media Cybernetics, Inc., USA). The average green fluorescence intensity of each pixel was normalized to the background intensity in the same image.

### RNA interference

SOCS3 siRNA and control siRNA were purchased from Santa Cruz Biotechnology (Santa Cruz, CA, USA). SOCS3 siRNA is a pool of three target-specific 19–25 nt siRNAs: (1) sense CAGCAUCUUUGUCGGAAGATT and antisense UCUUCCGACAAAGAUGCUGTT, (2) sense GUAUGAUGCUCCACUUUAATT and antisense UUAAAGUGGAGCAUCAUACTT, and (3) sense CCAAGUGUUGAACUUAGAATT and antisense UUCUAAGUUCAACACUUGGTT. Control siRNA was used as a negative control. 3.3 nmol siRNA was dissolved in 330 μL RNase-free water. For the transfection of siRNA, BV-2 cells were cultured in six-well plates with antibiotic-free medium the day before transfection. The transfection was conducted when cells reached 50~70% confluence using Lipofectamine 2000 (Invitrogen, USA) and serum-free medium according to the manufacturer’s instructions. After 4 h, the transfection medium was replaced with the culture medium containing 10% FBS and then incubated at 37 °C in 5% CO_2_.

### Intracellular calcium level measurement

BV-2 cells grown in 96-well plates were used for intracellular calcium level ([Ca^2+^]_i_) measurements in vitro. The growth medium was removed and replaced with dye loading buffer (80 μL/well) containing 4 μM Fluo-4/Fluo-2 and 0.5% bovine serum albumin in Locke’s buffer consisting of (in mM) 4-(2-hydroxyethyl)-1-piperazineethanesulfonic acid (8.6), KCl (5.6), NaCl (154), glucose (5.6), MgCl_2_ (1.0), CaCl_2_ (2.3), and glycine (0.1), pH 7.4. After 45 min of incubation in the dye loading buffer, the cells were washed four times in fresh Locke’s buffer (200 μL/well). For dynamic measurement, the cells were transferred to a fluorescence laser plate reader (FLIPR Tetra; Molecular Devices, Sunnyvale, CA, USA) incubation chamber. Different concentrations of lidocaine were added to the wells after a 2-min baseline recording from a compound plate in a volume of 25 μL using an automatic robotic system. The emitted fluorescence signals were recorded at 512–520 nm after excitation at 488 nm. For long period measurement, BV-2 cells were treated with different concentrations of lidocaine. The fluorescence signals were recorded at 515 nm after excitation at 490 nm by an end-point method using a Cytation™ 5 Cell Imaging Multi-Mode Reader (BioTek, USA).

### Quantitative PCR

Total RNA was extracted from BV-2 cells using TRIzol reagent (Invitrogen, CA, USA). Isolated RNA was reverse transcribed into cDNA using PrimeScript™ RT Reagent Kit (TaKaRa, Japan) following standard protocols. Real-time quantitative PCR (qPCR) was performed with synthetic primers and SYBR Green (TaKaRa, Japan) with a QuantStudio 5 Real-Time PCR Detection System (Thermo Fisher Scientific). The relative expression levels on *Socs3*, *Il1b*, and *Tnfa* were calculated and quantified with the 2^−ΔΔCt^ method after normalization with the reference *Gapdh* expression. All primers used are listed in Table 1.

**Table 1:** Sequences of primers for real-time quantitative polymerase chain reaction

Gene	Sequence
*Gapdh*	
Forward	5′-GGCATGGACTGTGGTCATGAG-3′
Reverse	5′-TGCACCACCAACTGCTTAGC-3′
*Il1b*	
Forward	5′-TCATTGTGGCTGTGGAGAAG-3′
Reverse	5′-AGGCCACAGGTATTTTGTCG-3′
*Tnfa*	
Forward	5′-CATCTTCTCAAAATTCGAGTGACAA-3′
Reverse	5′-TGGGAGTAGACAAGGTACAACCC-3′
*Socs3*	
Forward	5′-GCTCCAAAAGCGAGTACCAGC-3′
Reverse	5′-AGTAGAATCCGCTCTCCTGCAG-3′

*Gapdh* glyceraldehyde 3-phosphate dehydrogenase, *Il1b* interleukin-1β, *Tnfa* tumor necrosis factor-α, *Socs3* suppressor of cytokine signaling 3

### Measurements of cyclic adenosine monophosphate

Intracellular cyclic adenosine monophosphate (cAMP) was performed using cAMP ELISA kit (MSK, China) according to the manufacturer’s instruction. Briefly, BV-2 cells were grown in six-well plates. The culture medium was discarded, and the cells were washed once with PBS. Then, cells were harvested followed by repeated freeze-thaw to release intracellular components. The supernatants were measured by ELISA to assess the level of cAMP.

### Statistical analysis

GraphPad Prism 6 software (GraphPad Software, San Diego, CA, USA) was used to conduct all the statistical analyses. The differences between two groups were evaluated by Student’s *t* test. The data from more than two groups were evaluated by one-way ANOVA followed by Tukey’s multiple comparisons test or two-way ANOVA followed by Bonferroni post hoc tests. Results were represented as mean ± SEM of the independent experiments. Results described as significant were based on a criterion of *p* < 0.05.

## Results

### Lidocaine potentiates acute morphine analgesic effect and attenuates chronic morphine tolerance

**Fig. 1:**
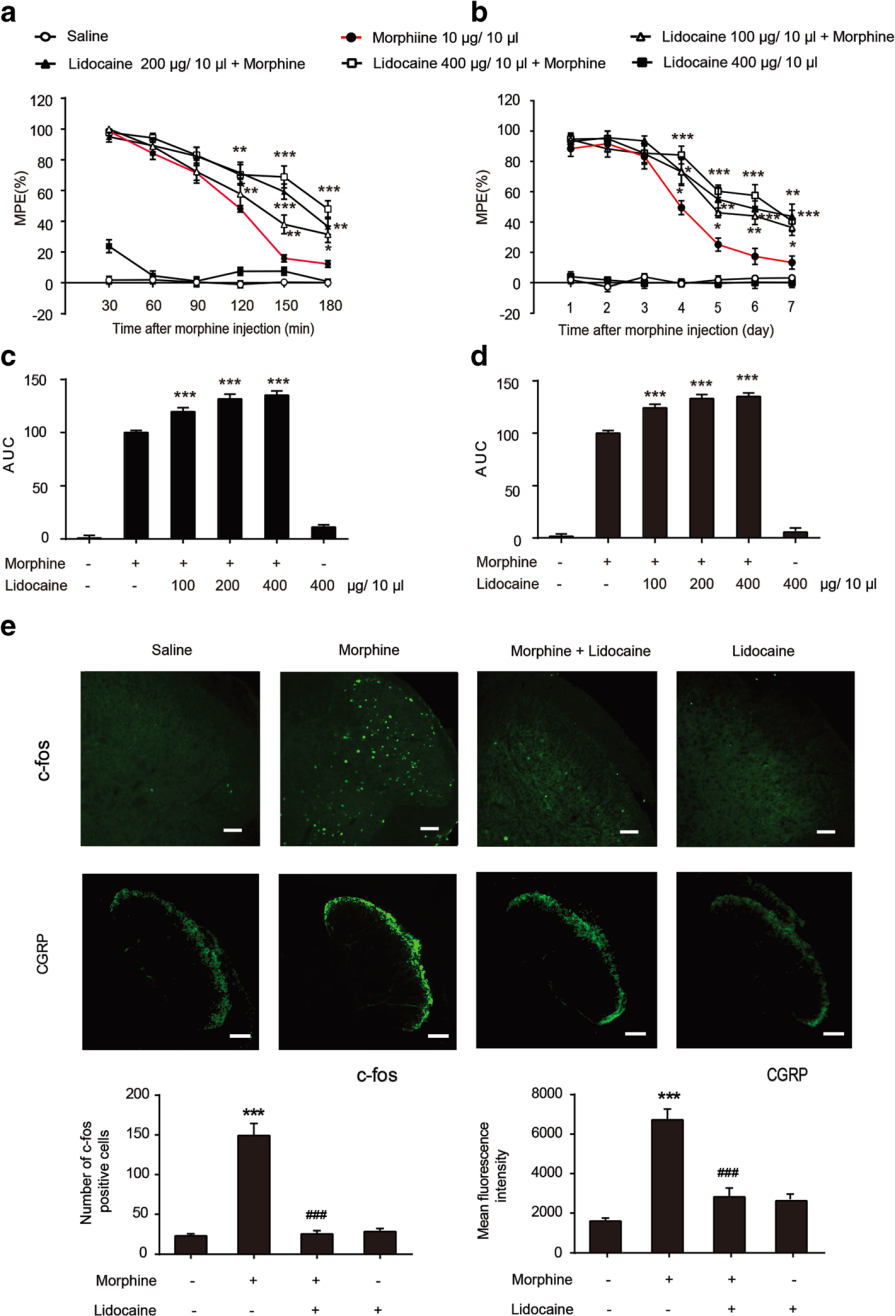
Lidocaine potentiates acute morphine analgesic effect and suppresses chronic morphine tolerance. The tail-flick test was performed to evaluate the effect of lidocaine on the morphine tolerance. Data were shown as a percentage of MPE. **a**, **c** Lidocaine co-administration with morphine potentiated acute morphine analgesic effect. Morphine (10 μg/10 μL, i.t.) with or without lidocaine (100, 200, and 400 μg/10 μL) was injected into mice, and analgesia was assessed at the first day (*n* = 8). **b**, **d** Lidocaine co-administration with morphine suppressed chronic morphine tolerance. Morphine (10 μg/10 μL) was intrathecally injected with different doses of lidocaine (100, 200, and 400 μg/10 μL) once daily, and the MPE was measured 1 h after the first injection of each day (*n* = 8). **e** Immunofluorescence images and analysis showed the activation of c-Fos and CGRP after morphine administration in the spinal cord. The quantification of c-Fos and CGRP immunofluorescence were respectively represented as the number of c-Fos-positive cells and mean fluorescence intensity of CGRP in the dorsal horns (*n* = 4). Lidocaine (100, 200, and 400 μg/10 μL, i.t.) was administered once daily for 7 days. After the final administration, spinal samples were collected. *p < 0.05, **p < 0.01, and ***p < 0.001 versus the saline group; ^###^ *p* < 0.001 versus the morphine-treated group. Scale bar 75 μm

We measured the latency of tail withdraw on mice by tail-flick assay to assess the analgesic effect of morphine. Lidocaine (up to 400 μg/10 μL) did not alter the pain threshold after 1 h of its administration (Additional file 1: Figure S1). Lidocaine significantly potentiated acute morphine analgesic effect and attenuated chronic morphine tolerance (Fig. 1a, b). At day 7, the MPE at 1 h after morphine administration decreased to 12.56% in chronic morphine-treated mice, whereas mice co-administrated with lidocaine (100, 200, and 400 μg/10 μL) and morphine displayed the MPE of 33.08, 39.57, and 36.94%, respectively (Fig. 1b). We also calculated the area under the curve (AUC) for acute analgesic effect (0–180 min) and for chronic analgesic effect (0–7 days) after morphine administration. We found that lidocaine increased the AUC of acute analgesic effect (0–180 min) and chronic analgesic effect (0–7 days) (Fig. 1c, d). Immunofluorescence staining data revealed a suppressive effect of lidocaine on the nociceptive-related activation of c-Fos and CGRP in the dorsal horn of the spinal cord after morphine administration (Fig. 1e).

### Lidocaine suppresses morphine-induced activation of microglia and attenuates the levels of proinflammatory cytokines in the spinal cord

**Fig. 2:**
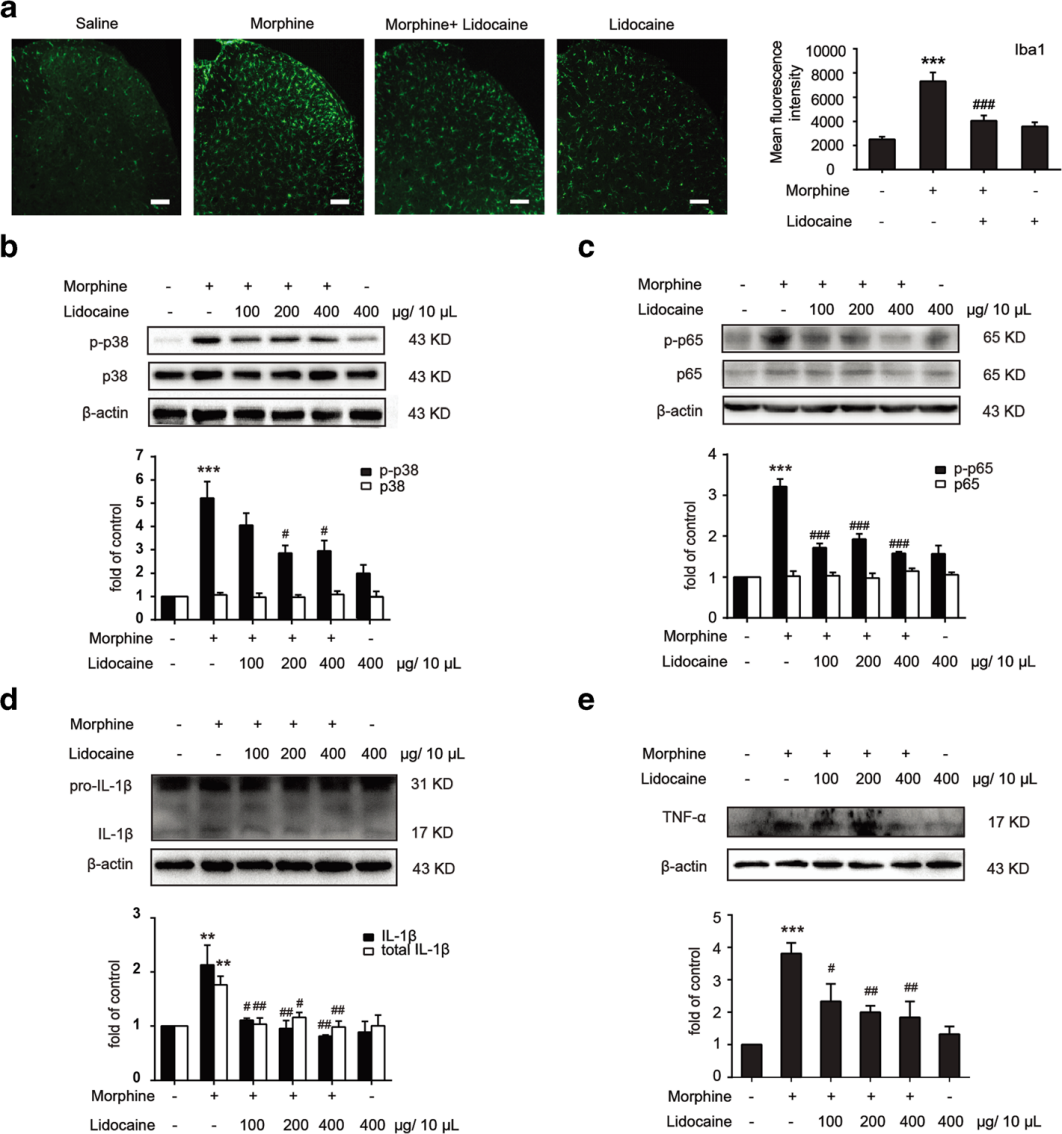
Lidocaine significantly attenuates chronic morphine-induced microglial activation and suppresses neuroinflammation-related proinflammatory cytokines in the spinal cord. **a** Immunofluorescence images and analysis showed 200 μg/10 μL lidocaine nearly completely inhibited the activation of microglia evoked by morphine in the spinal cord (*n* = 4). **b** Immunoblot results demonstrated that lidocaine inhibited morphine-induced upregulation of phosphorylation of p38 MAPK, but not the p38 total protein (*n* = 4). **c** Lidocaine markedly inhibited morphine-induced upregulation of p-p65 (*n* = 4). **d**, **e** Immunoblot results showed that the administration of lidocaine decreased the levels of mature IL-1β, total IL-1β, and TNF-α evoked by morphine. Lidocaine (100, 200, and 400 μg/10 μL, i.t.) was administered once daily for 7 days (*n* = 4). After the final administration, spinal samples were collected. **p < 0.01 and ***p < 0.001 versus the saline group; ^#^ *p* < 0.05, ^##^ *p* < 0.01, and ^###^ *p* < 0.001 versus the morphine-treated group. Scale bar 75 μm

Accumulating evidences indicate that neuroinflammation contributes to central sensitization after chronic morphine treatment through microglial activation. Specifically, IL-1β and TNF-α are the most important proinflammatory cytokines [24] reported in the development of morphine tolerance. Our immunofluorescence staining data showed that repeated morphine treatment (10 μg/10 μL, once daily for 7 days) resulted in the activation of the microglia (Iba1 as a microglia marker), and 200 μg/10 μL lidocaine co-administration with morphine nearly completely inhibited the activation of microglia (Fig. 2a). Furthermore, immunoblot result demonstrated that lidocaine remarkably suppressed the morphine-induced phosphorylation of p38 mitogen-activated protein kinase (MAPK) and p65 in the spinal cord (Fig. 2b, c). Then, we tested the effects of lidocaine on the proinflammatory cytokines upregulated during morphine tolerance. Immunoblot result showed that the administration of lidocaine decreased the level of mature IL-1β and total IL-1β induced by morphine (Fig. 2d), and lidocaine also significantly inhibited morphine-evoked upregulation of TNF-α in the spinal cord (Fig. 2e). These findings are consistent with a previous study, which demonstrated that lidocaine decreased the lipopolysaccharide (LPS)-induced activation of p38 MAPK along with the reduced level of proinflammatory cytokines [25].

### Lidocaine specially increases the level of SOCS3 in microglia of the spinal cord

**Fig. 3:**
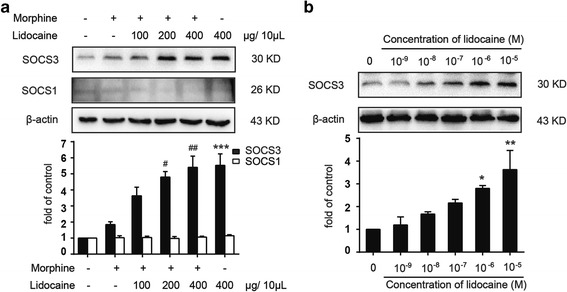
Lidocaine specially induces the upregulation of SOCS3. **a** Lidocaine resulted in an enhanced level of SOCS3 in the spinal cord without affecting SOCS1. Lidocaine (100, 200, and 400 μg/10 μL, i.t.) was intrathecally administered once daily for 7 days. After the final administration, spinal samples were collected (*n* = 4). **b** Lidocaine (0.001 to 10 μM) promoted the expression of SOCS3 in a concentration-dependent manner in BV-2 cells. (Data were obtained from three independent experiments). Cells were collected and analyzed 12 h or the indicated time after lidocaine treatment. *p < 0.05, **p < 0.01, and ***p < 0.001 versus the saline or vehicle group; ^#^ *p* < 0.05 and ^##^ *p* < 0.01 versus the morphine-treated group

**Fig. 4:**
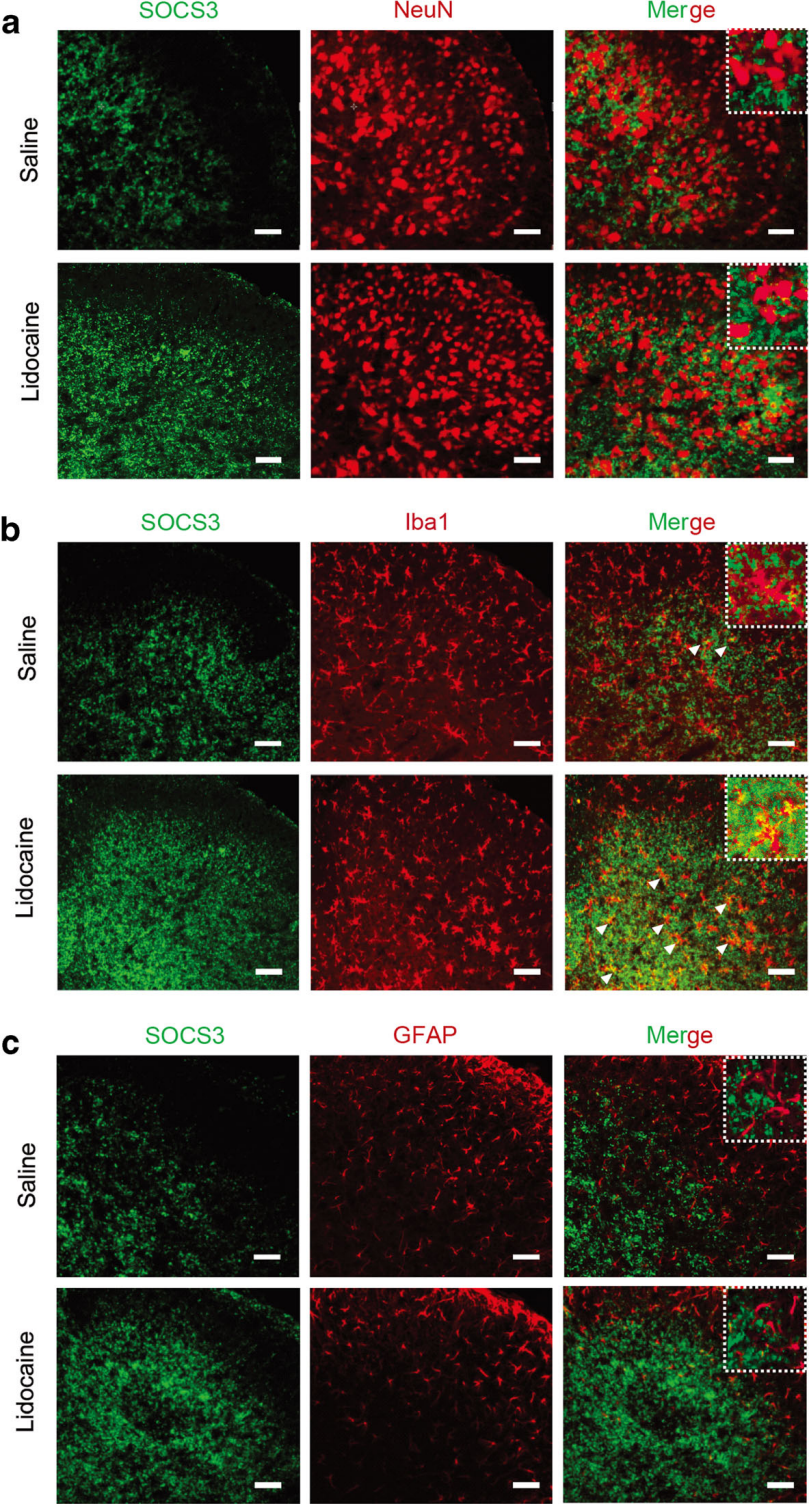
Distribution and cellular localization of SOCS3 in the dorsal horn of the spinal cord. **a** Confocal microscopy study for SOCS3 (green) and its co-localization with neurons (NeuN, red) in the dorsal horn. **b** Confocal microscopy study for SOCS3 (green) and its co-localization with microglia (Iba1, red) in the dorsal horn. **c** Confocal microscopy study for SOCS3 (green) and its co-localization with astrocytes (GFAP, red) in the dorsal horn. Lidocaine (100, 200, and 400 μg/10 μL, i.t.) was given once daily for 7 days. Spinal samples were collected after the last administration of lidocaine

Morphine-induced activation of TLR4-NF-κB signal pathway played an important role in the development of morphine tolerance. SOCS3 acted as an endogenous negative regulator that could suppress the downstream effects of TLR4 signal pathway and inhibit corresponding inflammation. In this study, we found that lidocaine specially induced the upregulation of SOCS3 in the spinal cord but not affected the level of SOCS1 (Fig. 3a). To confirm whether lidocaine was capable of inducing the expression of SOCS3 in vitro, immortalized murine microglial cell line BV-2 was utilized [26]. BV-2 cells were treated with different concentrations of lidocaine (from 0.001 to 10 μM) for 12 h. Immunoblot results showed that lidocaine significantly increased the expression of SOCS3 in a concentration-dependent manner in vitro (Fig. 3b). Furthermore, analysis of the cellular distribution of SOCS3 by confocal microscopic scanning showed that, in naive and lidocaine-treated mice, SOCS3 mainly co-localized with Iba1 (microglia marker), but not with NeuN (neuronal marker) or GFAP (astrocyte marker) in the spinal cord (Fig. 4a–c).

### The anti-inflammatory effect of lidocaine to morphine-induced cytokines is SOCS3 dependent in microglia

**Fig. 5:**
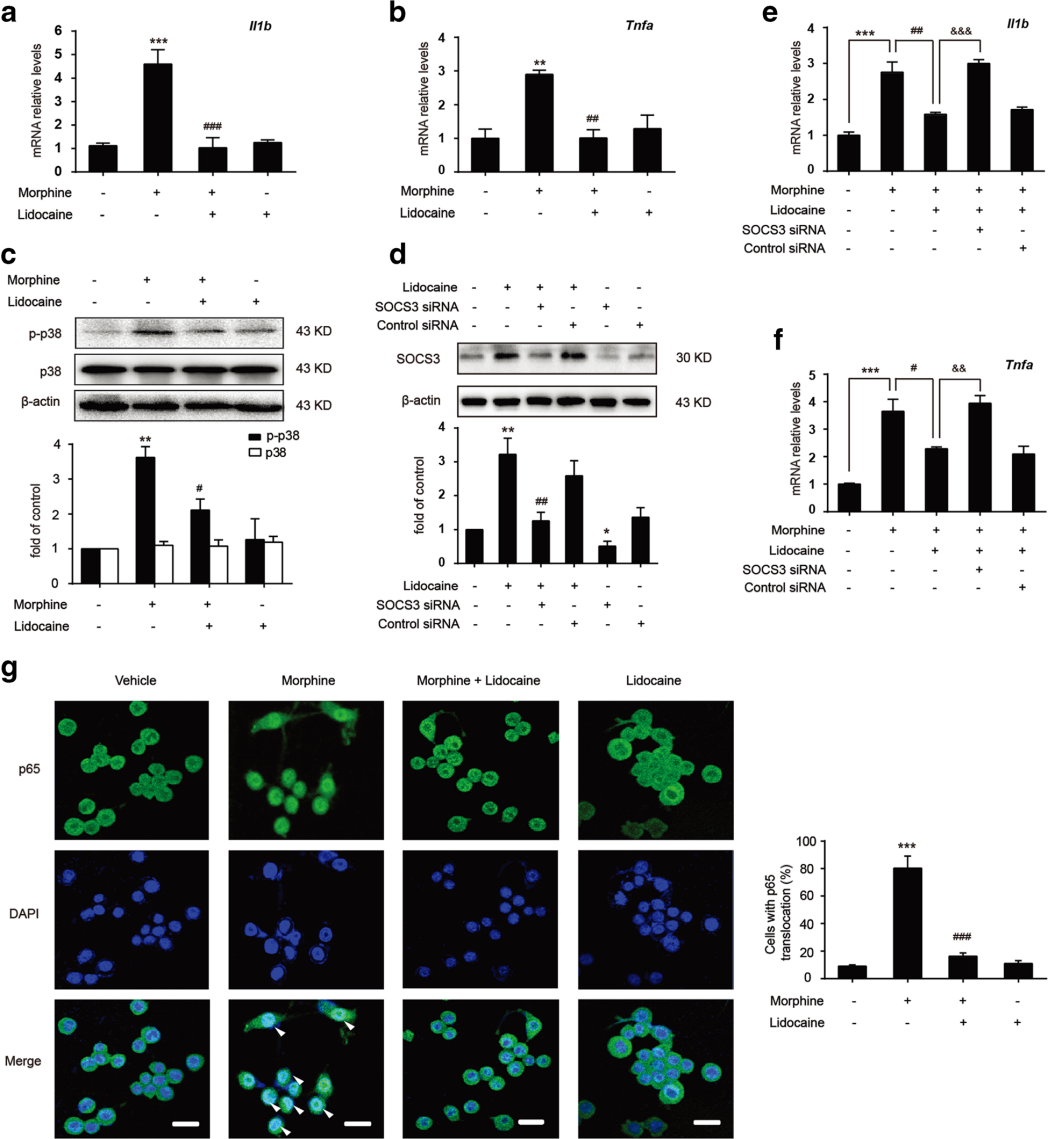
Lidocaine significantly inhibits morphine-induced inflammatory responses via a SOCS3-dependent way in BV-2 cells. **a**, **b** Real-time PCR showed suppressive effects of lidocaine on *Il1b* and *Tnfa* mRNA levels in morphine-stimulated BV-2 cells. Cells were pretreated with lidocaine (10 μM) for 12 h, followed by morphine (200 μM) treatment. Then, the cells were collected and analyzed 12 h after morphine treatment. **c** Effect of lidocaine on the phosphorylation of p38 MAPK in morphine-stimulated BV-2 cells. Cells were treated with lidocaine (10 μM) for 12 h before morphine (200 μM) treatment. **d** BV-2 cells were transfected with 100 pmol SOCS3 siRNA or control siRNA for 18 h, followed by 10 μM lidocaine treatment for 12 h. The efficiency of SOCS3 knockdown was assessed by immunoblot assay. **e**, **f** SOCS3 siRNA sufficiently abolished the anti-inflammatory effects of lidocaine on *Il1b* and *Tnfa* mRNA in BV-2 cells. BV-2 cells were transfected with 100 pmol SOCS3 siRNA or control siRNA and then subjected to 10 μM lidocaine pretreatment for 12 h, followed by exposure to morphine (200 μM) for 12 h. (**a**–**f** Data were obtained from three independent experiments). **g** Lidocaine (10 μM) inhibited the NF-κB translocation from the cytosol to the nucleus after morphine (200 μM) exposure for 1 h in BV-2 cells (*n* = 4). NF-κB activity was analyzed by p65 nuclear translocation assay. **p < 0.01 and ***p < 0.001 versus the vehicle group; ^#^ *p* < 0.05, ^##^ *p* < 0.01, and ^###^ *p* < 0.001 versus the morphine-treated group or lidocaine-treated group; ^&&^ *p* < 0.01 and ^&&&^ *p* < 0.001 versus the morphine and lidocaine-coadministered group. Scale bar 30 μm

Numerous evidences have shown that acute or chronic blockade of inflammatory signaling by various IL-1 blockers [27] and TNF-α inhibitors [14] significantly potentiated morphine analgesic effect and decreased the activation of microglia in the spinal cord. We studied the effects of lidocaine on morphine-induced microglial activation in vitro. Morphine (200 μM, 12 h) induced robust inflammatory responses in BV-2 cells, which were characterized by increasing levels of transcription for IL-1β and TNF-α (Fig. 5a, b). Real-time PCR showed suppressive effects of lidocaine (10 μM, 12 h) on IL-1β and TNF-α messenger RNA (mRNA) levels in morphine-stimulated BV-2 cells. Besides, morphine increased the phosphorylation of p38 MAPK (Fig. 5c) and caused the translocation of p65 NF-κB from the cytoplasm to the nucleus (Fig. 5g). Lidocaine (10 μM, 12 h) treatment markedly suppressed phosphorylation of p38 MAPK and inhibited the NF-κB translocation from the cytosol to the nucleus in morphine-stimulated BV-2 cells (Fig. 5c, g). In addition, we investigated whether the anti-inflammatory effects of lidocaine were SOCS3 dependent. SOCS3 small interfering RNA was utilized to downregulated SOCS3 (Fig. 5d). We found that the knockdown of SOCS3 sufficiently abolished anti-inflammatory effects of lidocaine (Fig. 5e, f). It demonstrated that SOCS3 was essential to the anti-inflammatory effects of lidocaine.

### Lidocaine-induced SOCS3 upregulation is calcium-dependent protein kinase kinase β-AMPK dependent

**Fig. 6:**
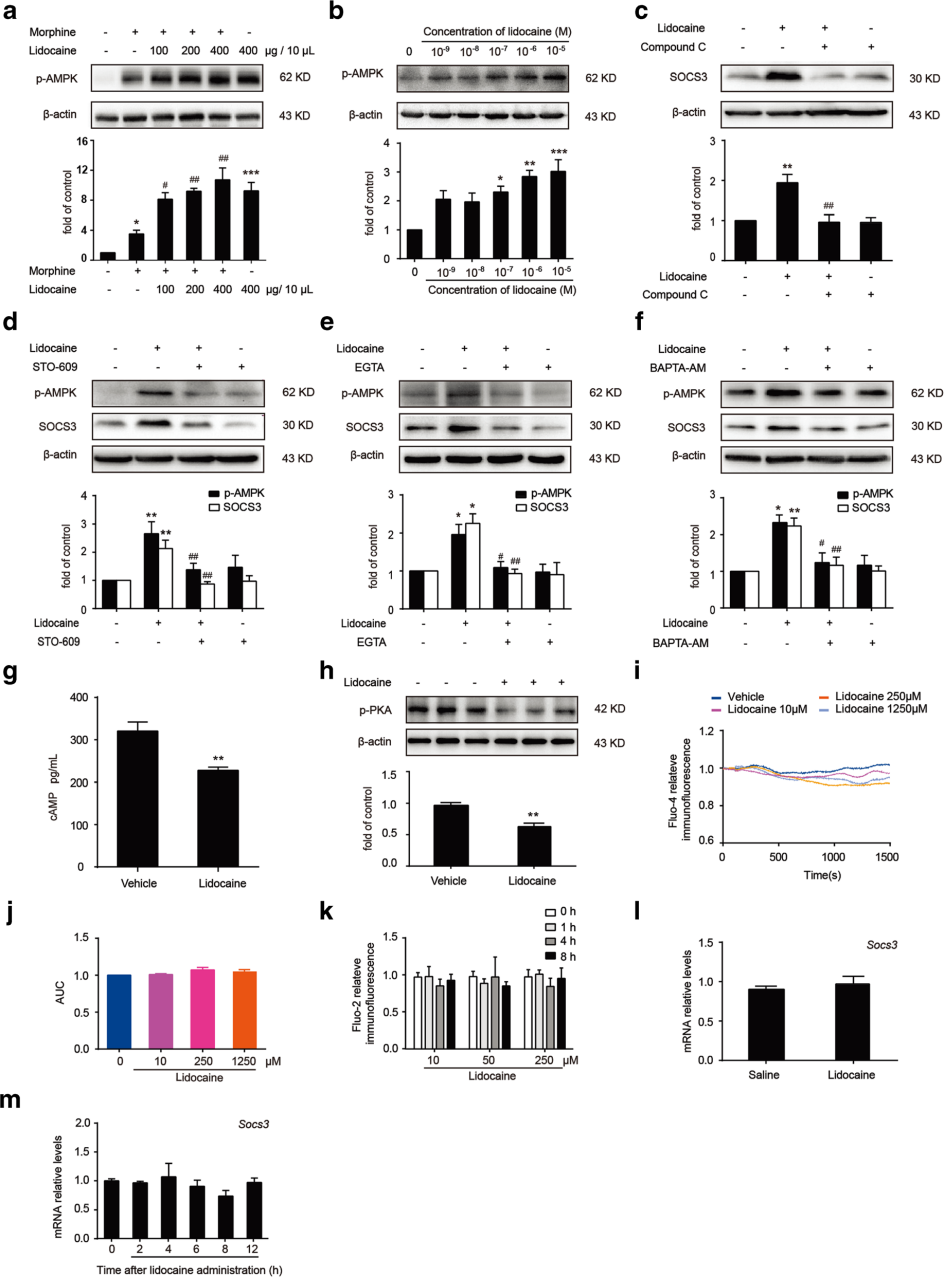
CaMKKβ-dependent AMPK activation is required in the lidocaine-mediated SOCS3 upregulation. **a** Lidocaine increased the levels of SOCS3 in the spinal cord. Lidocaine (100, 200, and 400 μg/10 μL, i.t.) was intrathecally administered once daily for 7 days. After the final administration, spinal samples were collected and evaluated by immunoblot assay (*n* = 4). **b** Lidocaine (0.001 to 10 μM) promoted phosphorylation of AMPK in a concentration-dependent manner in BV-2 cells. Cells were collected and analyzed 12 h after lidocaine treatment. **c** Compound C (20 μM) administration reversed lidocaine-induced upregulation of SOCS3. **d** STO-609 administration (20 μM, 2 h) prior to lidocaine administration (10 μΜ, 12 h) prevented the lidocaine-induced AMPK phosphorylation and SOCS3 expression. **e**, **f** Extracellular Ca^2+^ depletion with EGTA (2 mM) or intracellular Ca^2+^ depletion with BAPTA-AM (10 μM) abolished the increasing of SOCS3 and AMPK phosphorylation induced by lidocaine. BV-2 cells were pretreated with Ca^2+^ chelator for 30 min, followed by 10 μM lidocaine treatment for 12 h. **g** Lidocaine markedly inhibited the cAMP levels in BV-2 cells. Cells were collected and analyzed 12 h after lidocaine treatment (*n* = 6). **h** Lidocaine markedly inhibited the phosphorylation of PKA in BV-2 cells. Cells were collected and analyzed 12 h after lidocaine treatment. **i**, **j** Representative Ca^2+^ tracings and group data showed that lidocaine had no marked effect on [Ca^2+^]_i_ within 1500 min in BV-2 cells. **k** Lidocaine had no marked effect on [Ca^2+^]_i_ in BV-2 cells at 1, 4, and 8 h after treatment. **l**, **m** Lidocaine had no effect on *Socs3* mRNA in vivo (*n* = 3) and in vitro. (**b**–**f**, **h**–**k**, **m** Data were obtained from three independent experiments). *p < 0.05, **p < 0.01, and ***p < 0.001 versus the vehicle group; ^#^ *p* < 0.05 and ^##^ *p* < 0.01 versus the morphine-treated group or lidocaine-treated group

AMPK, a metabolic sensitive serine/threonine protein kinase, is increasingly recognized to play a central role in the regulation of neuroinflammation and pathogenesis of CNS diseases [28, 29]. It has been shown that AMPK activators can significantly inhibit inflammation in various model systems [30, 31] and the activation of AMPK significantly suppressed microglia activation by promoting M2 polarization [32]. It was also reported that SOCS3 deficiency promoted M1 polarization and induced inflammation [33, 34]. Furthermore, bupivacaine, a local anesthetic similar with lidocaine, was reported to be an activator of AMPK [23]. Considering all the factors above, we hypothesize that lidocaine inhibits neuroinflammation and improves morphine tolerance via AMPK-mediated upregulation of SOCS3. Immunoblot results showed that intrathecal administration of lidocaine significantly increased phosphorylation of AMPK at Thr172 in the spinal cord (Fig. 6a). We also found the phosphorylation of AMPK was significantly elevated after lidocaine (from 0.001 to 10 μM, 12 h) administration in a concentration-dependent manner in BV-2 cells (Fig. 6b). We then questioned whether AMPK was required for a lidocaine-induced increase of SOCS3. As expected, compound C (20 μM) significantly reversed lidocaine-induced upregulation of SOCS3 (Fig. 6c). The activation of AMPK is under the control of AMPKs such as calcium-dependent protein kinase kinase β (CaMKKβ) and tumor suppressor liver kinase B1 (LKB1). It was reported that CaMKKβ was crucial in the brain and CaMKKβ-dependent AMPK activation was an important mechanism underlying H_2_S suppression on neuroinflammation [35]. Therefore, we investigated whether the activation of AMPK is induced by lidocaine depending on CaMKKβ. STO-609 (20 μM), a CaMKK inhibitor, significantly suppressed the lidocaine-induced phosphorylation of AMPK (Fig. 6d). Similarly, STO-609 significantly suppressed the lidocaine-induced upregulation of SOCS3 (Fig. 6d). We also found that extracellular Ca^2+^ depletion with EGTA (2 mM) or intracellular Ca^2+^ depletion with BAPTA-AM (10 μM) abolished the increasing of SOCS3 and AMPK phosphorylation induced by lidocaine (Fig. 6e, f). It suggested that Ca^2+^ was essential to the regulation of SOCS3. Shen et al. reported that chronic administration of opioid agonist increased the level of cAMP and consequently activated protein kinase A (PKA) [36]. CaMKKβ was under the control of PKA [37]. Therefore, we investigated the effects of lidocaine on cAMP and PKA. The ELISA data demonstrated that lidocaine downregulated the level of cAMP (Fig. 6g), and immunoblot results indicated that lidocaine markedly inhibited the phosphorylation of PKA in BV-2 cells (Fig. 6h). It was in accordance with the previous study [38]. We also measured [Ca^2+^]_i_ within 1500 s after lidocaine administration and [Ca^2+^]_i_ for a long period up to 8 h. However, [Ca^2+^]_i_ was not changed by lidocaine (Fig. 6i–k). It suggested that Ca^2+^ was essential to the signal transduction caused by lidocaine, but not acted as a trigger.

Furthermore, we investigated the transcriptional level of SOCS3 after the administration of lidocaine. Interestingly, there was no difference between the control group and lidocaine-treated group in vivo and in vitro (Fig. 6l, m). It implied that post-transcriptional regulation to SOCS3 may be involved in the lidocaine-induced upregulation.

## Discussion

**Fig. 7:**
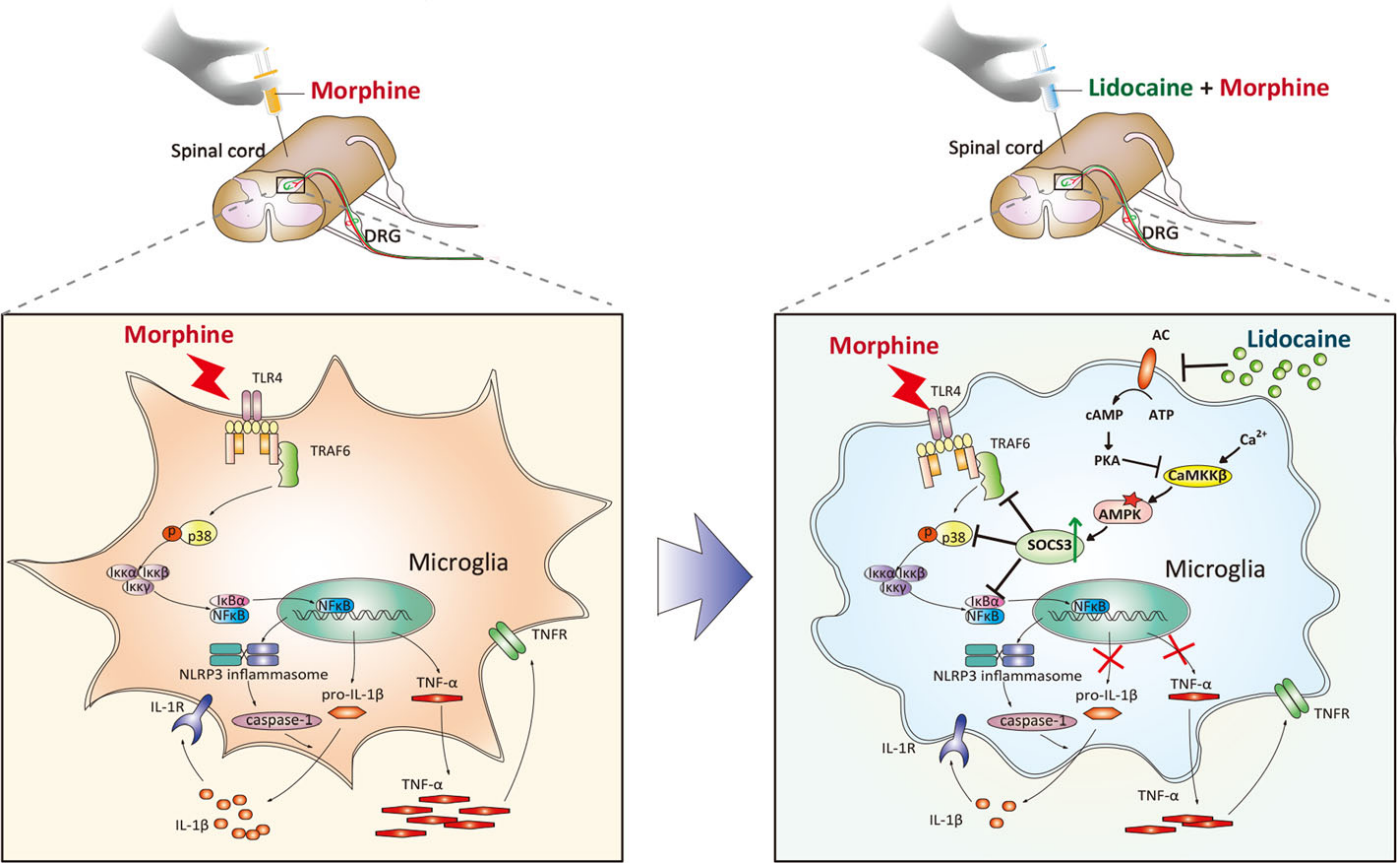
Schematic model indicates the AMPK-SOCS3-induced inflammation suppression improves morphine tolerance by lidocaine. Morphine induces activation of microglia and upregulation of proinflammatory cytokines including IL-1β and TNF-α via the TLR4/p38/NF-κB pathway. CaMKKβ-AMPK-dependent upregulation of SOCS3 in the spinal cord by lidocaine plays a crucial role in the suppression of microglia and improvement of morphine tolerance

In this study, we demonstrated that lidocaine markedly inhibited the development of chronic morphine tolerance and had a significant inhibitory effect on morphine-induced activation of microglia and consequent neuroinflammation; second, we illuminated that the improvement of morphine tolerance achieved by lidocaine was based on its upregulation of the level of SOCS3 via AMPK-dependent signal pathway (Fig. 7).

A growing body of literature implicated MAPK family and transcription factor NF-κB signaling pathway were intimately associated with morphine-induced neuroinflammation and anti-nociception tolerance. Their common upstream regulator is TLR4, which is generally expressed on microglia. Accumulating evidences showed that TLR4-mediated neuroinflammation in the spinal cord was critically involved in the development and maintenance of morphine tolerance [4, 39]. Studies demonstrated that systemic antagonism of TLR4 improved morphine tolerance. Inhibition of TLR4 gene expression [40] or blockade of TLR4 downstream signals both led to a marked potentiation of the magnitude and duration of opioid analgesia [41]. Unfortunately, there is no safe and effective TLR4 inhibitor available in clinic for the treatment of morphine tolerance. In addition, evidence showed that TAK1 served as a key molecule involved in the cascade that linked TLR4 to MAPK/NF-κB in this signal pathway and OZ, an inhibitor of TAK1, could prevent the development of tolerance to morphine-induced anti-nociception and reverse established tolerance [42]; however, it was not available in clinic. Yoshimura et al. reported TAK1 was negatively regulated by SOCS3, an endogenous anti-inflammatory protein [16]. In addition, therapeutic trials using SOCS3-specific anti-sense oligonucleotides, small hairpin RNAs, or cell-penetrating SOCS3 proteins have been performed [43].

Lidocaine, the most prevalent, effective local anesthetic drug, was reported to have anti-hyperalgesia and anti-inflammatory effects. In different disease models, intravenous administration of lidocaine reduced levels of TNF-α and IL-1β [44, 45]. However, the molecular mechanism was not fully understood. In Chinese clinic, it could be intrathecally administrated as a combination analgesic with morphine to treat severe pain at the concentration of 0.5–2.5% (equivalent to 50–250 μg/10 μL). Our study provided the direct evidences that lidocaine had obvious anti-tolerance effect in mice at the dosage of 100, 200, and 400 μg/10 μL (Fig. 1a, b). Consistent with our results, it was reported that systemic administration of lidocaine reduced morphine requirements and postoperative pain of patients undergoing thoracic surgery after propofol-remifentanil-based anesthesia [46]. In this study, we first time illuminated that lidocaine could inhibit morphine-induced neuroinflammation by upregulating SOCS3 via AMPK both in vivo and in vitro (Fig. 3a, b).

A recent study reported that in dorsal horn neurons, the binding of morphine to the opioid receptor activated phosphatidylinositol 3-kinase (PI3K)/Akt signaling, leading to the activation of mammalian target of rapamycin (mTOR) and resulting in the adaptive changes in protein translations in the spinal cord dorsal horn [2]. AMPK as an important kinase plays a critical role in cellular energy homeostasis, and its activation could inhibit mTORC1 activity [47]. Our previous studies revealed that AMPK was also involved in the regulation of neuroinflammation and the AMPK activators, metformin and resveratrol, could be utilized to improve morphine tolerance by inhibiting microglial-mediated neuroinflammation [48, 49]. In this study, we found that lidocaine increased the phosphorylation of AMPK in vivo (Fig. 6a). Furthermore, in BV-2 cells, various concentrations of lidocaine (from 0.001 to 10 μM, 12 h) led to the activation of AMPK in a concentration-dependent manner (Fig. 6b). Benkwitz had reported lidocaine could facilitate the ability of activated Gα_i_ to inhibit adenylate cyclase (AC) and finally decreased the level of cAMP [38]. Signals increasing the intracellular level of cAMP could activate PKA-dependent pathways and, in turn, negatively regulate CaMKKβ activity [37]. Our ELISA data indicated that lidocaine could downregulate the level of cAMP (Fig. 6g), and immunoblot results showed that lidocaine markedly inhibited the phosphorylation of PKA in BV-2 cells (Fig. 6h). It is reported that CaMKKβ and LKB1 are two critical kinases that could phosphorylate and activate AMPK [50]. In mammals, CaMKKβ is crucial in the brain and CaMKKβ-dependent AMPK activation is an important mechanism underlying H_2_S suppression on neuroinflammation, whereas LKB1 is expressed in multiple peripheral tissues such as liver, small intestine, and skeletal muscles [51]. Immunoblot analysis indicated that STO-609, a CaMKKβ inhibitor, reversed the increasing level of p-AMPK and SOCS3 induced by lidocaine (Fig. 6d). Similarly, compound C also broadly abrogated the effects caused by lidocaine (Fig. 6c). As mentioned above, lidocaine downregulated the level of cAMP and phosphorylation of PKA and, therefore, markedly suppressed the negative regulation of PKA to CaMKKβ. Altogether, our data demonstrated that lidocaine could be utilized to improve morphine tolerance by suppressing neuroinflammation via CaMKKβ-AMPK-dependent upregulation of SOCS3 in the spinal cord. Then, we evaluated the level of mRNA for *Socs3* after lidocaine treatment, and data showed that lidocaine had no effect on *Socs3* mRNA in vivo and in vitro (Fig. 6l, m). Based on our results mentioned above, lidocaine upregulated SOCS3 protein but not mRNA, and it suggested that post-transcriptional effects may be involved, such as microRNA. Lidocaine probably decreased the level of special microRNA targeting SOCS3, finally leading to the upregulation of SOCS3.

Our results indicated that lidocaine significantly inhibited morphine-induced activation of microglia and decreased the phosphorylation of p38 MAPK and NF-κB p65 in the spinal cord (Fig. 2b, c). Lidocaine also inhibited morphine-induced translocation of NF-κB p65 from the cytosol to the nucleus (Fig. 5g) and suppressed the level of IL-1β and TNF-α following morphine treatment (Fig. 2d, e). Furthermore, our study indicated that lidocaine decreased the level of CGRP, which was a peptide released by a primary afferent and was able to mediate the activation of NMDA receptors in neurons [52]. Lidocaine also downregulated c-Fos, which was implicated in pain transmission and morphine tolerance [15] (Fig. 1e). Therefore, lidocaine is an effective agent to improve morphine tolerance.

## Conclusions

In conclusion, we provided the evidence for the first time that lidocaine could extend acute morphine analgesia effect and improve morphine tolerance with a mechanism of inhibiting neuroinflammation (Fig. 7). Our data revealed that lidocaine relieved the activation of microglia and further decreased proinflammatory cytokines via CaMKKβ-AMPK-dependent upregulation of SOCS3 in the spinal cord (Fig. 7). Numerous evidences have shown that lidocaine had apparent anti-inflammatory effects and was utilized in the treatment of distal colitis, acute lung injury, and atopic dermatitis; however, the mechanism of lidocaine was not clear [53]. In this study, we proposed an explanation that associated lidocaine with AMPK-SOCS3. Our findings may represent a bright prospect for the improvement of morphine tolerance with lidocaine and lay the groundwork for treatment of patients with chronic pain.

## Additional file

Additional file 1: Figure S1. Lidocaine has no analgesic effect at 1 h after intrathecal administration. Mice were intrathecally injected with lidocaine (100, 200, 400 μg/10 μL) and analgesic effect was assessed at 0–120 min. Tail-flick method was performed to evaluate the analgesic effect of lidocaine. Data were shown as percentage of MPE (*n* = 8). ^**^ *p* < 0.01, ^***^ *p* < 0.001 versus saline group. (PDF 715 kb)

## Abbreviations

AC: Adenylate cyclase
AMPK: Adenosine 5′-monophosphate-activated protein kinase
AUC: Area under the curve
CaMKKβ: Calcium-dependent protein kinase kinase β
cAMP: Cyclic adenosine monophosphate
CGRP: Calcitonin gene-related peptide
CNS: Central nervous system
DAPI: 4,6-Diamino-2-phenylindole
EGTA: Ethylene glycol tetraacetic acid
FITC: Fluorescein isothiocyanate
GFAP: Glial fibrillary acidic protein
i.t.: Intrathecal
Iba1: Ionized calcium-binding adapter molecule 1
IL-1β: Interleukin-1β
LKB1: Liver kinase B1
LPS: Lipopolysaccharide
MAPK: Mitogen-activated protein kinase
MOR: Micro-opioid receptor
MPE: Maximal potential effect
mTOR: Mammalian target of rapamycin
NeuN: Neuronal nuclear protein
NF-κB: Nuclear factor-κB
NMDA: *N*-Methyl-d-aspartic acid
PBS: Phosphate-buffered saline
PI3K: Phosphatidylinositol 3-kinase
PKA: Protein kinase A
PKC: Protein kinase C
RIPA: Radio immunoprecipitation assay
siRNA: Small interfering RNA
SOCS1: Suppressor of cytokine signaling 1
SOCS3: Suppressor of cytokine signaling 3
TAK1: TGF-β-activated kinase 1
TLR4: Toll-like receptor 4
TNF-α: Tumor necrosis factor-α
TRAF6: TNF receptor-associated factor 6

## References

[1] Parvizpour A, Charkhpour M, Habibi-asl B, Shakhsi M, Ghaderi M, Hassanzadeh K (2013). Repeated central administration of selegiline attenuated morphine physical dependence in rat. Pharmacol Rep.

[2] Xu JT, Zhao JY, Zhao X, Ligons D, Tiwari V, Atianjoh FE, Lee CY, Liang L, Zang W, Njoku D (2014). Opioid receptor-triggered spinal mTORC1 activation contributes to morphine tolerance and hyperalgesia. J Clin Invest.

[3] Hassanzadeh BHAK (2004). Effects of ketamine and midazolam on morphine induced dependence and tolerance in mice. Daru Journal of Pharmaceutical Sciences.

[4] Hutchinson MR, Shavit Y, Grace PM, Rice KC, Maier SF, Watkins LR (2011). Exploring the neuroimmunopharmacology of opioids: an integrative review of mechanisms of central immune signaling and their implications for opioid analgesia. Pharmacol Rev.

[5] Williams JT, Ingram SL, Henderson G, Chavkin C, von Zastrow M, Schulz S, Koch T, Evans CJ, Christie MJ: Regulation of mu-opioid receptors: desensitization, phosphorylation, internalization, and tolerance. Pharmacol Rev 2013, 65:223–254. 10.1124/pr.112.005942 PMC3565916 23321159

[6] Fukagawa H, Koyama T, Kakuyama M, Fukuda K (2013). Microglial activation involved in morphine tolerance is not mediated by Toll-like receptor 4. J Anesth.

[7] Eidson LN, Inoue K, Young LJ, Tansey MG, Murphy AZ (2017). Toll-like receptor 4 mediates morphine-induced neuroinflammation and tolerance via soluble tumor necrosis factor signaling. Neuropsychopharmacology.

[8] Charkhpour M, Ghavimi H, Ghanbarzadeh S, Yousefi B, Khorrami A, Mesgari M, Hassanzadeh K (2015). Protective effect of pioglitazone on morphine-induced neuroinflammation in the rat lumbar spinal cord. J Biomed Sci.

[9] Horng T, Barton GM, Flavell RA, Medzhitov R (2002). The adaptor molecule TIRAP provides signalling specificity for Toll-like receptors. Nature.

[10] Kagan JC, Medzhitov R (2006). Phosphoinositide-mediated adaptor recruitment controls Toll-like receptor signaling. Cell.

[11] Lehnardt S, Massillon L, Follett P, Jensen FE, Ratan R, Rosenberg PA, Volpe JJ, Vartanian T (2003). Activation of innate immunity in the CNS triggers neurodegeneration through a Toll-like receptor 4-dependent pathway. Proc Natl Acad Sci U S A.

[12] Olson JK, Miller SD (2004). Microglia initiate central nervous system innate and adaptive immune responses through multiple TLRs. J Immunol.

[13] Latremoliere A, Woolf CJ (2009). Central sensitization: a generator of pain hypersensitivity by central neural plasticity. J Pain.

[14] Eidson LN, Murphy AZ (2013). Blockade of Toll-like receptor 4 attenuates morphine tolerance and facilitates the pain relieving properties of morphine. J Neurosci.

[15] Mattioli TA, Leduc-Pessah H, Skelhorne-Gross G, Nicol CJ, Milne B, Trang T, Cahill CM (2014). Toll-like receptor 4 mutant and null mice retain morphine-induced tolerance, hyperalgesia, and physical dependence. PLoS One.

[16] Yoshimura A, Naka T, Kubo M (2007). SOCS proteins, cytokine signalling and immune regulation. Nat Rev Immunol.

[17] Mahony R, Ahmed S, Diskin C, Stevenson NJ (2016). SOCS3 revisited: a broad regulator of disease, now ready for therapeutic use?. Cell Mol Life Sci.

[18] Tetzlaff JE (2000). The pharmacology of local anesthetics. Anesthesiol Clin North Am.

[19] Suzuki N, Hasegawa-Moriyama M, Takahashi Y, Kamikubo Y, Sakurai T, Inada E (2011). Lidocaine attenuates the development of diabetic-induced tactile allodynia by inhibiting microglial activation. Anesth Analg.

[20] Caracas HC, Maciel JV, Martins PM, de Souza MM, Maia LC: The use of lidocaine as an anti-inflammatory substance: a systematic review. J Dent 2009, 37:93–97. 10.1016/j.jdent.2008.10.005 19058888

[21] Jiao Q, Wang H, Hu Z, Zhuang Y, Yang W, Li M, Yu X, Liang J, Guo Y, Zhang H (2013). Lidocaine inhibits staphylococcal enterotoxin-stimulated activation of peripheral blood mononuclear cells from patients with atopic dermatitis. Arch Dermatol Res.

[22] De Oliveira GS, Fitzgerald P, Streicher LF, Marcus RJ, McCarthy RJ: Systemic lidocaine to improve postoperative quality of recovery after ambulatory laparoscopic surgery. Anesth Analg 2012, 115:262–267. 10.1213/ANE.0b013e318257a380 22584558

[23] Lu J, Xu SY, Zhang QG, Lei HY (2011). Bupivacaine induces reactive oxygen species production via activation of the AMP-activated protein kinase-dependent pathway. Pharmacology.

[24] Cai Y, Kong H, Pan YB, Jiang L, Pan XX, Hu L, Qian YN, Jiang CY, Liu WT (2016). Procyanidins alleviates morphine tolerance by inhibiting activation of NLRP3 inflammasome in microglia. J Neuroinflammation.

[25] Cunningham C (2013). Microglia and neurodegeneration: the role of systemic inflammation. Glia.

[26] Jeong H-J, Lin D, Li L, Zuo Z (2012). Delayed treatment with lidocaine reduces mouse microglial cell injury and cytokine production after stimulation with lipopolysaccharide and interferon γ. Anesth Analg.

[27] Shavit Y, Wolf G, Goshen I, Livshits D, Yirmiya R (2005). Interleukin-1 antagonizes morphine analgesia and underlies morphine tolerance. Pain.

[28] Amato S, Man HY (2011). Bioenergy sensing in the brain: the role of AMP-activated protein kinase in neuronal metabolism, development and neurological diseases. Cell Cycle.

[29] Li J, McCullough LD (2010). Effects of AMP-activated protein kinase in cerebral ischemia. J Cereb Blood Flow Metab.

[30] Bai A, Ma AG, Yong M, Weiss CR, Ma Y, Guan Q, Bernstein CN, Peng Z (2010). AMPK agonist downregulates innate and adaptive immune responses in TNBS-induced murine acute and relapsing colitis. Biochem Pharmacol.

[31] Nath N, Khan M, Paintlia MK, Singh I, Hoda MN, Giri S (2009). Metformin attenuated the autoimmune disease of the central nervous system in animal models of multiple sclerosis. J Immunol.

[32] Xu Y, Xu Y, Wang Y, Wang Y, He L, Jiang Z, Huang Z, Liao H, Li J, Saavedra JM (2015). Telmisartan prevention of LPS-induced microglia activation involves M2 microglia polarization via CaMKKβ-dependent AMPK activation. Brain Behav Immun.

[33] Qin H, Holdbrooks AT, Liu Y, Reynolds SL, Yanagisawa LL, Benveniste EN (2012). SOCS3 deficiency promotes M1 macrophage polarization and inflammation. J Immunol.

[34] Qin H, Yeh WI, De Sarno P, Holdbrooks AT, Liu Y, Muldowney MT, Reynolds SL, Yanagisawa LL, Fox TH, 3rd, Park K, et al: Signal transducer and activator of transcription-3/suppressor of cytokine signaling-3 (STAT3/SOCS3) axis in myeloid cells regulates neuroinflammation. Proc Natl Acad Sci U S A 2012, 109:5004–5009. 10.1073/pnas.1117218109 PMC3323949 22411837

[35] Zhou X, Cao Y, Ao G, Hu L, Liu H, Wu J, Wang X, Jin M, Zheng S, Zhen X (2014). CaMKKbeta-dependent activation of AMP-activated protein kinase is critical to suppressive effects of hydrogen sulfide on neuroinflammation. Antioxid Redox Signal.

[36] Shen J, Benedict Gomes A, Gallagher A, Stafford K, Yoburn BC (2000). Role of cAMP-dependent protein kinase (PKA) in opioid agonist-induced mu-opioid receptor downregulation and tolerance in mice. Synapse.

[37] Racioppi L, Means AR (2012). Calcium/calmodulin-dependent protein kinase kinase 2: roles in signaling and pathophysiology. J Biol Chem.

[38] Benkwitz C, Garrison JC, Linden J, Durieux ME, Hollmann MW (2003). Lidocaine enhances Galphai protein function. Anesthesiology.

[39] Shen CH, Tsai RY, Shih MS, Lin SL, Tai YH, Chien CC, Wong CS (2011). Etanercept restores the antinociceptive effect of morphine and suppresses spinal neuroinflammation in morphine-tolerant rats. Anesth Analg.

[40] Shafie A, Moradi F, Izadpanah E, Mokarizadeh A, Moloudi MR, Nikzaban M, Hassanzadeh K (2015). Neuroprotection of donepezil against morphine-induced apoptosis is mediated through Toll-like receptors. Eur J Pharmacol.

[41] Hutchinson MR, Zhang Y, Shridhar M, Evans JH, Buchanan MM, Zhao TX, Slivka PF, Coats BD, Rezvani N, Wieseler J (2010). Evidence that opioids may have Toll-like receptor 4 and MD-2 effects. Brain Behav Immun.

[42] Xu H, Xu T, Ma X, Jiang W (2015). Involvement of neuronal TGF-beta activated kinase 1 in the development of tolerance to morphine-induced antinociception in rat spinal cord. Br J Pharmacol.

[43] Carow B, Rottenberg ME (2014). SOCS3, a major regulator of infection and inflammation. Front Immunol.

[44] Peiro JR, Barnabe PA, Cadioli FA, Cunha FQ, Lima VM, Mendonca VH, Santana AE, Malheiros EB, Perri SH, Valadao CA (2010). Effects of lidocaine infusion during experimental endotoxemia in horses. J Vet Intern Med.

[45] Nishina K, Mikawa K, Maekawa N, Takao Y, Obara H (1995). Does early posttreatment with lidocaine attenuate endotoxin-induced acute injury in rabbits?. Anesthesiology.

[46] Cui W, Li Y, Li S, Wang R, Li J (2010). Systemic administration of lidocaine reduces morphine requirements and postoperative pain of patients undergoing thoracic surgery after propofol-remifentanil-based anaesthesia. Eur J Anaesthesiol.

[47] Inoki K, Kim J, Guan KL (2012). AMPK and mTOR in cellular energy homeostasis and drug targets. Annu Rev Pharmacol Toxicol.

[48] Pan Y, Sun X, Jiang L, Hu L, Kong H, Han Y, Qian C, Song C, Qian Y, Liu W (2016). Metformin reduces morphine tolerance by inhibiting microglial-mediated neuroinflammation. J Neuroinflammation.

[49] Han Y, Jiang C, Tang J, Wang C, Wu P, Zhang G, Liu W, Jamangulova N, Wu X, Song X (2014). Resveratrol reduces morphine tolerance by inhibiting microglial activation via AMPK signalling. Eur J Pain.

[50] Peng IC, Chen Z, Sun W, Li YS, Marin TL, Hsu PH, Su MI, Cui X, Pan S, Lytle CY (2012). Glucagon regulates ACC activity in adipocytes through the CAMKKbeta/AMPK pathway. Am J Physiol Endocrinol Metab.

[51] Korsse SE, Peppelenbosch MP, van Veelen W: Targeting LKB1 signaling in cancer. Biochimica Et Biophysica Acta-Reviews on Cancer 2013, 1835:194–210. 10.1016/j.bbcan.2012.12.006 23287572

[52] Yan H, Yu LC (2013). Expression of calcitonin gene-related peptide receptor subunits in cultured neurons following morphine treatment. Neurosci Lett.

[53] Bjorck S, Dahlstrom A, Ahlman H (2002). Treatment of distal colitis with local anaesthetic agents. Pharmacology & Toxicology.

