# Supplementary material for: Lidocaine alleviates morphine tolerance via AMPK-SOCS3-dependent neuroinflammation suppression in the spinal cord
Source: J Neuroinflammation. 2017 Nov 2;14:211. doi: 10.1186/s12974-017-0983-6 (PMC5667445; doi:10.1186/s12974-017-0983-6)
Supplement: Additional file 1: Figure S1. — Lidocaine has no analgesic effect at 1 h after intrathecal administration. Mice were intrathecally injected with lidocaine (100, 200, 400 μg/10 μL) and analgesic effect was assessed at 0–120 min. Tail-flick method was performed to evaluate the analgesic effect of lidocaine. Data were shown as percentage of MPE (n = 8). ** p < 0.01, *** p < 0.001 versus saline group. (PDF 715 kb) [file 12974_2017_983_MOESM1_ESM.pdf]

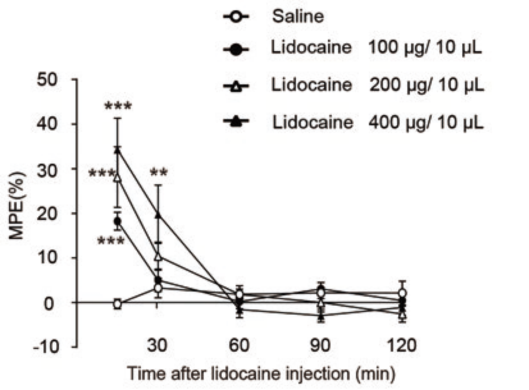
Saline
Lidocaine 100 μg/ 10 μL
Lidocaine 200 μg/ 10 μL
Lidocaine 400 μg/ 10 μL
50
40
30
20
10
0
-10
MPE(%)
***
***
**
***
30
60
90
120
Time after lidocaine injection (min)
